# Supplementary material for: Myoglobin in Brown Adipose Tissue: A Multifaceted Player in Thermogenesis
Source: Cells. 2023 Sep 8;12(18):2240. doi: 10.3390/cells12182240 (PMC10526770; doi:10.3390/cells12182240)
Supplement: Supplementary file 1 [file cells-12-02240-s001.zip › cells-2573764-supplementary.pdf]

*Supplementary Material*

# Myoglobin in Brown Adipose Tissue: A Multifaceted Player in Thermogenesis

**Mostafa A. Aboouf [1,2,3,*], Thomas A. Gorr [1], Nadia M. Hamdy [3], Max Gassmann [1,2] and Markus Thiersch [1,2]**

1 Institute of Veterinary Physiology, University of Zurich, 8057 Zurich, Switzerland
2 Zurich Center for Integrative Human Physiology (ZIHP), University of Zurich, 8057 Zurich, Switzerland
3 Department of Biochemistry, Faculty of Pharmacy, Ain Shams University, 11566 Cairo, Egypt
* Correspondence: mostafaaboouf.ali@uzh.ch; Tel.: +41-44-635-88-16

**Tabel S1.** Single Cell RNA-seq-based muscle cell transcriptome analyzed with regard to specificity, illustrating the number of genes with elevated expression in each specific muscle cell type compared to other cell types.

| Cell type | Tissue of origin | Function of elevated myoglobin | Cluster | Genes within cluster | # of genes elevated |
|---|---|---|---|---|---|
| **Cardiomyocytes** | Heart muscle | facilitates oxygen transport in muscles & contraction | - | 404 | 742 |
| **Skeletal myocytes** | Skeletal muscle | | 13 | 318 | 510 |
| **Smooth muscle cells** | Adipose tissue, Breast, Bronchus, Endometrium, Heart muscle, Liver, Lung, Ovary, Prostate, Skeletal muscle, Tongue, Vascular | Little Myoglobin is detected in smooth muscles | - | - | 374 |

The transcript profiling was based on publicly available genome-wide expression data from scRNA-seq experiments covering 29 tissues and peripheral blood mononuclear cells (PBMCs). All datasets (unfiltered read counts of cells) were clustered separately using louvain clustering, resulting in a total of 536 different cell type clusters https://www.proteinatlas.org/ENSG00000198125-MB.

https://www.proteinatlas.org/humanproteome/single+cell+type/muscle+cells#skeletalmyocytes (Accessed on June 1st, 2023).

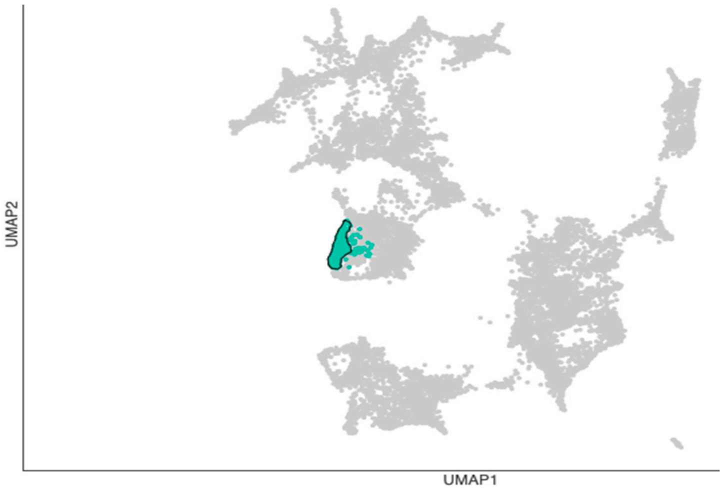


**A**

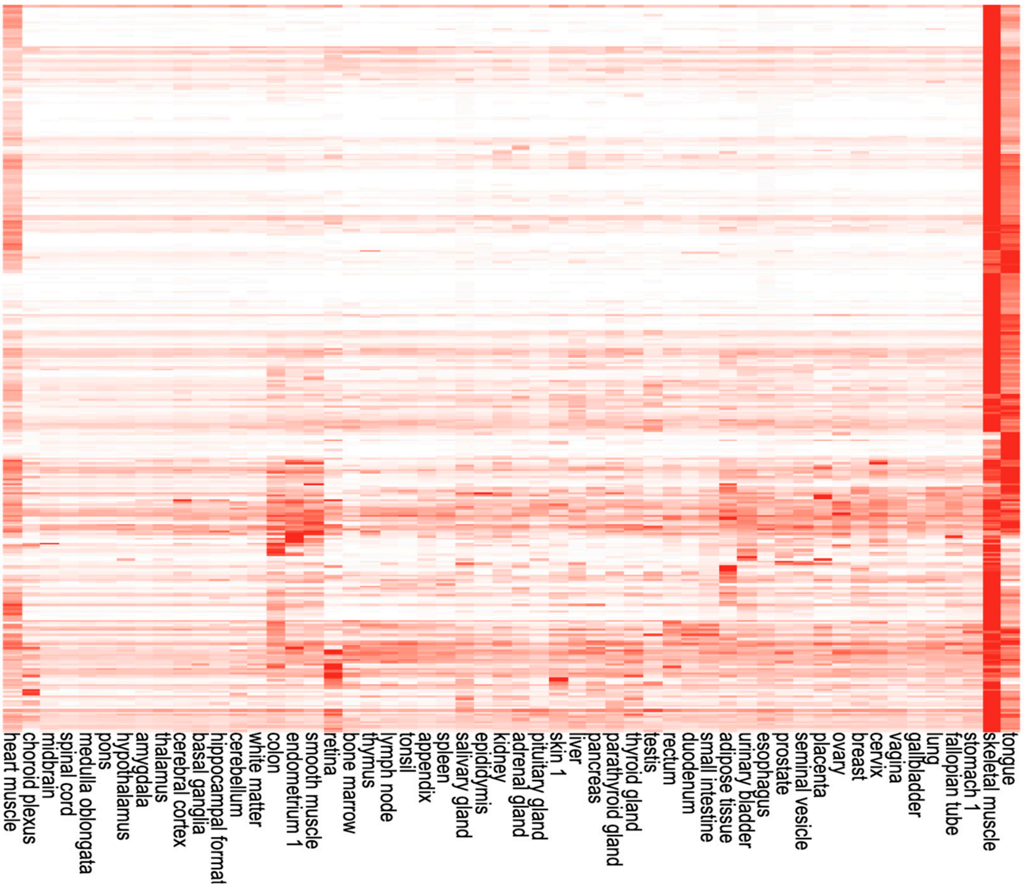


**B** **relative expression scale**

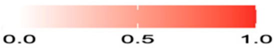


**Figure S1.** A) Myoglobin gene tissue expression cluster 13: striated muscle - muscle contraction (green colored with 318 genes within the cluster), B) Myoglobin gene within the 318 genes relative expression heatmap (relative expression scale from 0.0 as faint red-pink color for no expression to 1.0 red color as highly expressed in tongue and skeletal muscle the right-side of the heatmap as well as heart muscle the left-side of the heatmap). Organism homo sapiens, https://www.proteinatlas.org/humanproteome/tissue/expression+cluster#cluster13 (Accessed on June 1st, 2023).
